# Transient efficacy of buparvaquone against *Theileria haneyi* in chronically infected horses

**DOI:** 10.1186/s13071-024-06397-0

**Published:** 2024-08-12

**Authors:** Cynthia K. Onzere, Amany Hassan, Kelly Sears, Lowell S. Kappmeyer, Nicolas F. Villarino, Lindsay M. Fry, Reginaldo G. Bastos

**Affiliations:** 1 Department of Veterinary Microbiology and Pathology, Washington State University, Pullman, WA USA; 2 Department of Animal Medicine, Faculty of Veterinary Medicine, University of Alexandria, Alexandria, Egypt; 3 Carlson College of Veterinary Medicine, Oregon State University, Corvallis, OR USA; 4 Animal Disease Research Unit, USDA-ARS, Pullman, WA USA; 5 Department of Veterinary Clinical Sciences, College of Veterinary Medicine, Washington State University, Pullman, USA

**Keywords:** *Theileria haneyi*, Buparvaquone, Equine piroplasmosis, Hematological parameters, Blood urea nitrogen

## Abstract

**Background:**

*Theileria haneyi* is one of the three known causative agents of equine piroplasmosis. While imidocarb is generally effective in the clearance of the highly pathogenic *Theileria equi*, it is ineffective in the treatment of *T. haneyi*. Moreover, co-infection with *T. haneyi* has been shown to impede the successful treatment of *T. equi*. Furthermore, tulathromycin and diclazuril have demonstrated inefficacy in eradicating *T. haneyi*. The absence of an effective therapeutic agent against this parasite represents a significant obstacle in managing equine piroplasmosis.

**Methods:**

To address this issue, we evaluated the efficacy of buparvaquone in the treatment of *T. haneyi* in chronically infected horses.

**Results:**

Our findings showed that treatment of horses with the recommended dose of 2.5 mg/kg of buparvaquone led to a rapid abatement of *T. haneyi* levels, to a level where the parasites were not detectable by nested PCR. Following treatment, the horses remained PCR negative for a minimum of seven weeks until recrudescence occurred. Subsequent re-administration of buparvaquone at an increased dosage of 6 mg/kg upon recrudescence failed to exert a theilericidal effect on *T. haneyi*. Throughout the treatment regimen, the hematological parameters of the horses and most components of the chemistry panel remained within the normal range, except for blood urea nitrogen levels, which fell below the normal range in certain instances.

**conclusions:**

BPQ at 2.5 mg/kg and 6 mg/kg had a robust theilericidal effect but was ineffective in the clearance of the *T. haneyi* infection in persistently infected animals.

**Graphical Abstract:**

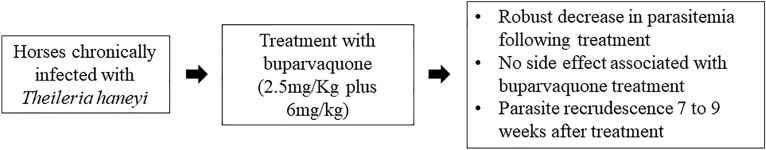

**Supplementary Information:**

The online version contains supplementary material available at 10.1186/s13071-024-06397-0.

## Background

Equine piroplasmosis is an acute hemolytic disease that affects all equid species [1]. The disease is caused by parasites that belong to the order Piroplasmida [2], including *Babesia caballi*, *Theileria equi* [1], and the recently discovered *Theileria haneyi* [3]. Equine piroplasmosis is associated with substantial economic losses in the equine industry due to restrictions on international exportation of animals, high cost of treatment, abortions, death, and poor animal performance [1, 4]. This negatively impacts livelihoods, especially in resource-poor countries [5].

*Theileria haneyi* was first reported near Eagle Pass, Texas, USA, and it was identified as a *Theileria* species due to its morphology and replication pattern [3]. The study showed that *T. haneyi* is distinct from *T. equi* in its 18S ribosomal deoxyribonucleic acid (rDNA) phylogeny, genome, predicted protein profile, immunological response, and significantly smaller size of merozoites when compared with *T. equi* [3]. The study also showed that even though *T. haneyi* leads to hemolysis in infected animals, and thus a reduction in hematocrit, the parasite infection does not appear to lead to severe clinical disease in spleen-intact horses like the highly virulent *T. equi* [3, 6]. However, the effect of *T. haneyi* infection on the performance of animals remains to be further investigated. In this regard, it is imperative to develop effective therapeutics for the treatment of both *T. haneyi* and *T. equi*.

To date, efforts to culture both the schizont and merozoite stages of *T. haneyi* have been unsuccessful, rendering it difficult to evaluate the effectiveness of therapeutic candidates in vitro. As a result, all studies have been conducted in vivo. For instance, a study conducted to investigate the efficacy of imidocarb dipropionate on *T. haneyi* infection in horses showed that the drug failed to clear the infection, and the parasite interfered with the efficacy of the drug in horses co-infected with *T. equi* [6]. Additionally, a study of the effectiveness of tulathromycin and diclazuril on *T. haneyi*-infected horses showed that both drugs were unable to clear the infection [7]. The lack of effective treatment poses a major challenge in the control of *T. haneyi*, and this is exacerbated by the lack of knowledge of tick species that transmit the parasite, making it difficult to predict the parasite’s epidemiology globally.

To determine whether readily available chemotherapeutics can be used in the treatment of *T. haneyi*, the effectiveness of buparvaquone (BPQ) was assessed in the treatment of the parasite. BPQ was selected due to its effectiveness in the treatment of clinical bovine theileriosis caused by the highly pathogenic *Theileria* species, i.e., *T. parva* and *T. annulata*.

The mechanism of action of BPQ involves suppression of the pre-schizont stages of infection and killing of schizonts and piroplasms by blocking the parasites’ mitochondrial electron transport system [8]. A study conducted on cattle that were naturally infected with *T. parva* showed that BPQ cleared the parasite in 90% of the treated animals [9]. A similar study conducted on *T. annulata* showed that BPQ was effective against the parasite at a dose of 2.5 mg/kg [10]. In other studies, even though BPQ was effective in the treatment of clinical theileriosis, the drug did not completely clear the parasites in some cases. A study conducted on *T. equi* revealed that treating chronically infected horses with 2.5 mg/kg BPQ four times at 96-h intervals resulted in the elimination of the parasite [11]. However, this finding could not be replicated with increased doses of 3.5 mg/kg and 5 mg/kg [11]. The study also showed that BPQ at doses of 4 to 6 mg/kg was effective in the treatment of clinical disease caused by *T. equi* in splenectomized ponies, but the animals remained carriers [11]. Similarly, treatment of horses four times with 5 mg/kg at 48-h intervals led to a rapid suppression of *T. equi*, but recrudescence was observed in the treated animals [12].

Considering the differences between *T. haneyi* and *T. equi* described above, in this study, we examined whether BPQ is more effective in the clearance of *T. haneyi* than has been demonstrated for *T. equi* [11, 12]. Therefore, the objective of this study was to investigate the efficacy of BPQ in treating horses chronically infected with *T. haneyi*, because spleen-intact animals do not exhibit severe clinical signs during acute experimental infection, thus invalidating the need for therapeutics during this phase of infection [3, 13]. Additionally, since *T. haneyi* leads to the establishment of a persistent infection characterized by the consistent presence of the parasite in peripheral circulation, these animals remain as reservoirs for tick and/or iatrogenic transmission, thus presenting challenges in the control of the parasite. In this regard, treatment of the parasite in chronically infected equids is key to obliteration of parasite transmission. Evaluation of BPQ in the clearance of *T. haneyi* in chronically infected horses and implications of the results for equine piroplasmosis are discussed in the present study.

## Methods

### Parasite

The *T. haneyi* Eagle Pass isolate was used in this study [3].

### Experimental animals

Seven Welsh ponies (HO-270, HO-364, HO-776, HO-777, HO-784, HO-411 and HO-413) that had been used in previous studies were reused in this study [6, 7]. Animals were free of *T. equi* and *B. caballi* infections and were 8 to 12 years old at the beginning of the experiment and kept in a tick-free environment throughout the study.

### Infection of horses

HO-270 was infected by transfusion with whole blood obtained from a horse that had tested positive for *T. haneyi*, as described previously [3, 6]. HO-364, HO-776, HO-777, and HO-784 were infected intravenously using 2–4 ml of *T. haneyi* blood stabilate at 8.8% parasitized erythrocytes (PPE) [6]. HO-411 and HO-413 were infected with the *T. haneyi* blood stabilate as described previously [7]. At the time of this study, all the animals remained in a chronic phase of *T. haneyi* infection, consistently testing polymerase chain reaction (PCR)-positive for the parasite for at least 2 years, and yet exhibited no symptoms.

### Treatment of horses and sample collection

HO-270, HO-364, HO-776, HO-777, and HO-784 (treatment [Tx] groups) were treated four times at 4-day intervals with BPQ (Buparvex; Bimeda, Nairobi, Kenya) at the manufacturer’s recommended dose of 2.5 mg/kg administered intramuscularly (IM). HO-411 and HO-413 were not treated and were used as controls. HO-270, HO-364, HO-776, and HO-777 were re-treated four times at 4-day intervals with 6 mg/kg of BPQ IM at 18 weeks after the first treatment. The horses were monitored for discomfort and pain during treatment, and no such side effects were observed. HO-784 was re-treated with BPQ at 19 weeks after the first treatment, after finishing treatment with a non-steroidal anti-inflammatory drug for an unrelated leg injury. Whole blood samples were collected from the animals in 10-ml BD Vacutainer^®^ tubes (Becton, Dickinson and Company, Franklin Lakes, NJ, USA) containing EDTA, and serum samples were collected in Vacutainer^®^ tubes without additives. In the treatment group, whole blood samples were collected from the animals prior to the initial treatment (week 0) and weekly after treatment. Serum samples were collected from week 0 to week 11 post-treatment with 2.5 mg/kg and at week 18 to week 31 post-treatment with the same dose. In the control group, whole blood was collected weekly from week 0 to week 9 post-treatment and at weeks 15, 19, and 27 post-treatment. Serum samples were collected weekly at 0 to 7 weeks post-treatment and at weeks 23 and 27 post-treatment.

### Evaluation of the presence of *T. haneyi* DNA in chronically infected horses

DNA extraction was performed on whole blood samples using the Qiagen DNeasy^®^ Blood & Tissue Kit (Qiagen, Hilden, Germany) in accordance with the manufacturer’s instructions. Nested PCR targeting a hypothetical gene syntenic to the *T. equi ema-1* gene was used for the detection of *T. haneyi* DNA, as described previously [6]. DNA obtained from a horse that had consistently tested positive for *T. haneyi* was used as a positive control, and sterile 1× phosphate-buffered saline (PBS) (Thermo Fisher Scientific, Waltham, MA, USA) was used as a negative extraction control. Sterile water was used as a no-template control. In addition to amplifying *T. haneyi* DNA, amplification of the equine β-actin gene was performed for assessment of the presence of amplifiable DNA using specific primers (equine β-actin forward (fwd) TGGCATCCACGAAACTACCT and equine β-actin reverse (rev) TCTGCTGGAAGGTGGACAAT) that target a 248-base-pair (bp) region. The DreamTaq Green PCR Master Mix (2×) (Thermo Fisher Scientific) was used to prepare the reaction mix, and the thermocycling conditions were as follows: initial denaturation at 95 °C for 3 min followed by 34 cycles of denaturation at 95 °C for 30 s, annealing at 55 °C for 30 s and extension at 72 °C for 30 s. All PCR products were analyzed using a 1.5% agarose gel, and the ChemiDoc™ Touch Imaging System (Bio-Rad, Hercules, CA, USA) was used for image acquisition and subsequent visualization.

### Evaluation of blood parameters, parasitemia, and pyrexia

Blood parameters including assessment of hematocrit/packed cell volume (PCV) were measured on 200 µl of whole blood collected in Vacutainer tubes containing ethylenediaminetetraacetic acid (EDTA) using the ProCyte Dx™ analyzer (IDEXX Laboratories, Inc., Westbrook, ME, USA) in accordance with the manufacturer’s instructions. The Catalyst One Veterinary Blood Chemistry Analyzer (IDEXX Laboratories, Inc.) was used for evaluation of the chemistry panel on serum samples as directed by the manufacturer. The IDEXX VetConnect PLUS software (IDEXX Laboratories) was used for visualization of data from the ProCyte Dx™ and the Catalyst One Veterinary Blood Chemistry Analyzer. Evaluation of parasitemia was performed on blood smears stained with the PROTOCOL™ Hema 3™ stain set (Fisher Healthcare, Houston, TX, USA), and PPE was determined using the formula (total parasites in 5 fields)/(erythrocyte count in 1/4 of a field × 20) × 100, as described previously [7]. Pyrexia was assessed weekly by measurement of the rectal temperature of the horses in degrees Celsius (°C). The horses were considered pyrexic if the temperature was greater than 38.3 °C.

### Horse splenectomy

Splenectomy was performed to suppress horse immunity with the expectation of increased parasite levels in cases where the parasite was below detectable levels in spleen-intact horses. This enabled us to determine whether BPQ completely cleared the infection in horses that had tested negative for more than 5 weeks after re-treatment with 6 mg/kg of the drug. HO-270 and HO-776 were splenectomized at 34 weeks post-treatment. Surgeries were performed at the Washington State University (WSU) Veterinary Teaching Hospital (VTH) as described previously [13]. Briefly, prior to surgery, horses were kept off feed for 24 h and water for 6–8 h, and anesthesia was performed by a veterinary anesthesiologist. An approximately 30-cm skin incision was made over the 16th rib beginning at the paralumbar muscles and extending distally to 5 cm ventral to the costochondral junction. The incision was extended through the subcutaneous tissues and muscles. A careful stab incision was then made through the periosteum and peritoneum. The apex of the spleen was exteriorized, exposing the gastrosplenic ligament and associated vessels which were ligated and transected. The spleen was partially elevated and shifted ventrally to access the nephrosplenic ligament. The ligament was bluntly and sharply dissected off the axial splenic surface. Phenylephrine was then injected directly into the spleen to cause splenic contraction. The hilus was double-ligated and cauterized, and the spleen was then removed. Post-surgery recovery followed standard procedures at WSU-VTH. Post-surgery pain was managed by the administration of non-steroidal anti-inflammatory drugs, at the recommended dosages. The animals were discharged from WSU-VTH 1 week after the surgery.

## Results

### BPQ has a transient theilericidal effect on *T. haneyi* in chronically infected horses

Nested PCR confirmed that all the animals in the treatment group were positive for *T. haneyi* infection prior to treatment (week 0 post-treatment) (Table 1). Administration of BPQ at a dose of 2.5 mg/kg effectively suppressed the parasite in the treatment group, as evidenced by all tested animals turning PCR-negative within 1 week of treatment (Table 1). The horses remained *T. haneyi*-negative for a minimum of 7 weeks until recrudescence of parasitemia was observed in HO-776, HO-777, and HO-784 at 8 weeks post-treatment (Table 1). Recrudescence was also observed in HO-364 at 9 weeks post-treatment and at 14 weeks post-treatment in HO-270 (Table 1). Interestingly, an increase in dosage to 6 mg/kg did not prevent recrudescence. Moreover, even though HO-270 and HO-776 tested negative for *T. haneyi* by PCR for more than 5 weeks after re-treatment with the increased dose, removal of the spleen led to detection of the parasites by PCR within a week after surgery (Table 1), confirming that the parasites were still present in the animals.

**Table 1:** Summary of *T. haneyi* nested PCR (nPCR) and equine β-actin PCR results during treatment (Tx) with BPQ

Weeks post-Tx	HO-270		HO-364		HO-776		HO-777		HO-784		HO-411		HO-413	
	*T. haneyi* nPCR	β-Actin PCR	*T. haneyi* nPCR	β-Actin PCR	*T. haneyi* nPCR	β-Actin PCR	*T. haneyi* nPCR	β-Actin PCR	*T. haneyi* nPCR	β-Actin PCR	*T. haneyi* nPCR	β-Actin PCR	*T. haneyi* nPCR	β-Actin PCR
0^a^	+	+	+	+	+	+	+	+	+	+	+	+	+	+
1	−	+	−	+	−	+	−	+	−	+	+	+	+	+
2	−	+	−	+	−	+	−	+	−	+	+	+	+	+
3	−	+	−	+	−	+	−	+	−	+	+	+	+	+
4	−	+	−	+	−	+	−	+	−	+	+	+	+	+
5	−	+	−	+	−	+	−	+	−	+	+	+	+	+
6	−	+	−	+	−	+	−	+	−	+	+	+	+	+
7	−	+	−	+	−	+	−	+	−	+	+	+	+	+
8	−	+	−	+	+	+	+	+	+	+	+	+	+	+
9	−	+	+	+	+	+	+	+	−	+	+	+	+	+
10	−	+	+	+	−	+	−	+	+	+	+	+	+	+
11	−	+	−	+	+	+	−	+	+	+	+	+	+	+
12	−	+	−	+	−	+	−	+	−	+	+	+	+	+
13	−	+	−	+	−	+	+	+	+	+	+	+	+	+
14	+	+	−	+	+	+	+	+	+	+	+	+	+	+
15	−	+	+	+	+	+	−	+	−	+	+	+	+	+
16	−	+	+	+	+	+	+	+	+	+	+	+	+	+
17	+	+	+	+	−	+	−	+	+	+	+	+	+	+
18^a^	−	+	+	+	−	+	−	+	−	+	+	+	+	+
19^b^	−	+	+	+	+	+	−	+	+	+	+	+	+	+
20	−	+	−	+	−	+	−	+	+	+	+	+	+	+
21	−	+	+	+	−	+	−	+	+	+	*nt*	*nt*	*nt*	*nt*
22	−	+	+	+	−	+	−	+	+	+	*nt*	*nt*	*nt*	*nt*
23	−	+	−	+	+	+	+	+	−	+	+	+	+	+
24	+	+	+	+	+	+	+	+	−	+	*nt*	*nt*	*nt*	*nt*
25	−	+	+	+	−	+	+	+	+	+	*nt*	*nt*	*nt*	*nt*
26	−	+	+	+	−	+	−	+	−	+	*nt*	*nt*	*nt*	*nt*
27	−	+	−	+	−	+	−	+	−	+	+	+	+	+
28	−	+	+	+	−	+	−	+	+	+	*nt*	*nt*	*nt*	*nt*
29	−	+	+	+	−	+	−	+	+	+	*nt*	*nt*	*nt*	*nt*
30	−	+	+	+	−	+	+	+	+	+	*nt*	*nt*	*nt*	*nt*
31	−	+	−	+	+	+	−	+	+	+	+	+	*nt*	*nt*
32	−	+	+	+	−	+	+	+	+	+	*nt*	*nt*	*nt*	*nt*
33	−	+	−	+	+	+	+	+	+	+	*nt*	*nt*	*nt*	*nt*
34	*spl*	*nt*	*nt*	*nt*	*spl*	*nt*	*nt*	*nt*	*nt*	*nt*	*nt*	*nt*	*nt*	*nt*
35	+	*nt*	*nt*	*nt*	+	*nt*	*nt*	*nt*	*nt*	*nt*	*nt*	*nt*	*nt*	*nt*

*spl* splenectomy, *nt* not tested

^a^Represents the time of treatment at 0 and 18 weeks

^b^Represents treatment of HO-784 at 19 weeks post-treatment with 6 mg/kg of BPQ

Unlike the untreated controls (HO-411 and HO-413), which consistently tested PCR-positive throughout the study, a cyclical pattern marked by alternating phases of positivity and negativity was observed in the treatment group throughout the study (Table 1). Notably, the cyclical patterns were unique to each treated animal (Table 1).

### Treatment of chronically infected horses with BPQ does not alter hematological parameters

The following hematological parameters were assessed during this study: red blood cells (RBC), hematocrit/PCV, hemoglobin, mean cell volume (MCV), mean corpuscular hemoglobin (MCH), mean corpuscular hemoglobin concentration (MCHC), red blood cell distribution width (RDW), platelets and white blood cells (WBC) including eosinophils, basophils, neutrophils, lymphocytes, and monocytes. BPQ did not alter any of the hematologic components over time at either 2.5 mg/kg or 6 mg/kg, since the parameter values were within normal range throughout the study in both the treated and untreated animals (data not shown).

Since *T. haneyi* causes hemolysis followed by reduced PCV in infected horses during acute infection, it is worth noting that PCV levels were maintained at normal range throughout the study in both the treated and untreated groups (Table 2). Additionally, *T. haneyi* were undetectable on blood smears (0% PPE) in both groups (Table 2), and pyrexia was not observed in any of the animals (Supplementary Table 1), indicating that the treatment and subsequent parasite relapse did not alter the horses’ asymptomatic status.

**Table 2:** Summary of PCV (%) and PPE in *T. haneyi*-infected horses during treatment (Tx) with BPQ

Weeks post-Tx	HO-270		HO-364		HO-776		HO-777		HO-784		HO-411		HO-413	
	PCV	PPE	PCV	PPE	PCV	PPE	PCV	PPE	PCV	PPE	PCV	PPE	PCV	PPE
0^a^	50.1	0	45.1	0	40.3	0	42.6	0	36.9	0	35.9	0	46.5	0
1	44.3	0	44.2	0	32.7	0	37.9	0	34.9	0	41.8	0	44.8	0
2	44.9	0	40.7	0	39.1	0	43.0	0	39.8	0	45.1	0	40.0	0
3	49.3	0	43.8	0	40.4	0	41.2	0	37.8	0	47.0	0	45.1	0
4	44.0	0	41.9	0	40.2	0	40.2	0	39.3	0	40.0	0	47.0	0
5	42.5	0	39.8	0	43.5	0	39.6	0	42.1	0	47.0	0	40.0	0
6	45.2	0	42.3	0	42.6	0	41.1	0	39.7	0	45.0	0	47.0	0
7	47.9	0	48.2	0	39.8	0	44.5	0	42.5	0	41.0	0	45.0	0
8	44.9	0	47.4	0	36.5	0	40.7	0	34.8	0	37.0	0	41.0	0
9	42.8	0	42.6	0	40.6	0	36.9	0	37.0	0	35.0	0	40.0	0
10	48.0	0	42.6	0	45.6	0	41.8	0	42.9	0	*nt*	0	*nt*	0
11	50.2	0	36.8	0	40.3	0	39.3	0	34.7	0	*nt*	0	*nt*	0
12	41.5	0	41.2	0	34.7	0	33.1	0	35.5	0	*nt*	0	*nt*	0
13	69.4	0	37.3	0	36.0	0	33.8	0	34.8	0	*nt*	0	*nt*	0
14	42.0	0	41.0	0	46.2	0	36.7	0	33.1	0	*nt*	0	*nt*	0
15	36.5	0	40.5	0	36.9	0	38.4	0	34.8	0	42.8	0	40.9	0
16	36.8	0	38.5	0	37.1	0	38.0	0	34.3	0	*nt*	0	*nt*	0
17	36.9	0	43.0	0	39.1	0	39.0	0	29.3	0	*nt*	0	*nt*	0
18^a^	37.6	0	38.6	0	38.1	0	42.2	0	33.1	0	*nt*	0	*nt*	0
19^b^	44.7	0	43.5	0	32.6	0	39.3	0	31.4	0	40.7	0	39.3	0
20	39.4	0	37.5	0	34.5	0	36.0	0	36.0	0	*nt*	*nt*	*nt*	0
21	41.3	0	41.3	0	43.9	0	37.6	0	38.1	0	*nt*	*nt*	*nt*	*nt*
22	42.4	0	42.4	0	33.5	0	36.7	0	34.4	0	*nt*	*nt*	*nt*	*nt*
23	43.0	0	43.0	0	36.2	0	36.1	0	37.6	0	40	0	33.6	0
24	40.6	0	40.6	0	47.9	0	44.8	0	47.4	0	*nt*	*nt*	*nt*	*nt*
25	51.0	0	54.1	0	42.9	0	40.3	0	40.5	0	*nt*	*nt*	*nt*	*nt*
26	43.8	0	49.3	0	45.3	0	44.3	0	47.8	0	*nt*	*nt*	*nt*	*nt*
27	48.1	0	52.7	0	44.4	0	43.2	0	42.4	0	38.5	0	40.1	0
28	46.9	0	48.7	0	36.3	0	41.4	0	39.6	0	*nt*	*nt*	*nt*	*nt*
29	47.0	0	48.9	0	41.7	0	44.3	0	43.5	0	*nt*	*nt*	*nt*	*nt*
30	45.0	0	43.7	0	46.6	0	38.5	0	44.8	0	*nt*	*nt*	*nt*	*nt*
31	43.4	0	51.3	0	43.9	0	39.3	0	37.1	0	*nt*	*nt*	*nt*	*nt*
32	41.9	0	49.3	0	41.8	0	42.3	0	39.7	0	*nt*	*nt*	*nt*	*nt*
33	48.6	0	41.6	0	37.6	0	38.7	0	41.3	0	*nt*	*nt*	*nt*	*nt*
34	*spl*	*nt*	*nt*	*nt*	*spl*	*nt*	*nt*	*nt*	*nt*	*nt*	*nt*	*nt*	*nt*	*nt*
35	35.8	*nt*	*nt*	*nt*	32.0	*nt*	*nt*	*nt*	*nt*	*nt*	*nt*	*nt*	*nt*	*nt*

*spl* splenectomy, *nt* not tested

^a^Represents the time of treatment at 0 and 18 weeks

^b^Represents treatment of HO-784 at 19 weeks post-treatment with 6 mg/kg of BPQ

### Horses treated with BPQ show a decline in blood urea nitrogen level

Evaluation of the horses’ chemistry panel included assessment of glucose, creatinine, blood urea nitrogen (BUN), phosphorous, calcium, total protein, albumin, globulin, albumin-to-globulin ratio, alanine aminotransferase (ALT), alkaline phosphatase (ALP), gamma-glutamyl transferase (GGT), bilirubin, and cholesterol.

**Fig. 1:**
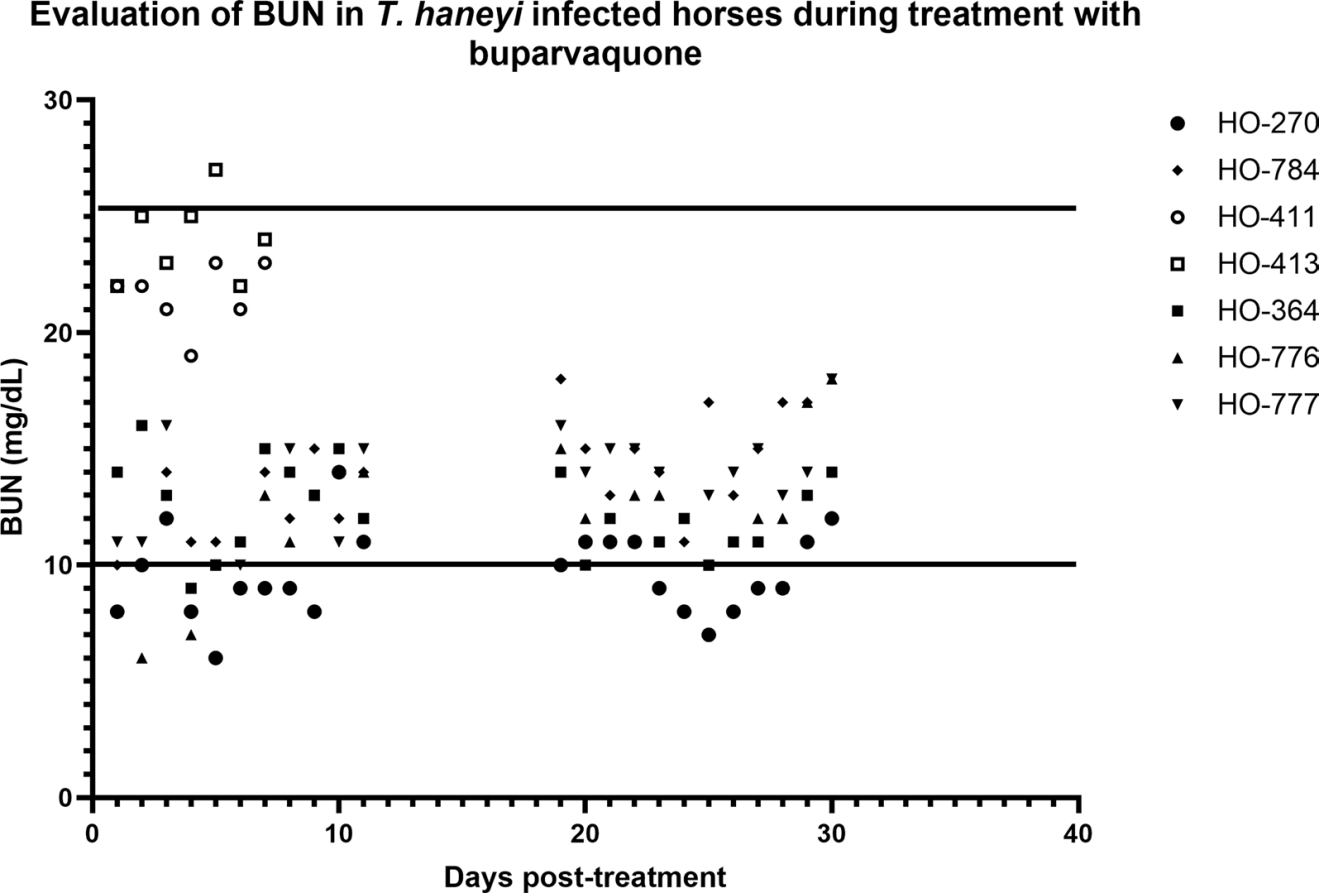
A decline in blood urea nitrogen (BUN) level is observed during treatment of chronically infected horses with BPQ. The two horizontal lines represent the normal range of BUN in horses (10–25 mg/dl)

No significant changes were observed in the chemistry panel of the horses before and after treatment (data not shown) except for BUN. After treatment with 2.5 mg/kg, a drastic decline in BUN levels was observed in HO-776 as early as 1 week post-treatment, and the values remained below the normal range until 2 weeks post-treatment before recovering (Fig. 1). In the case of HO-270, BUN fell below the normal range at 1 week post-treatment before returning to the normal range at 2 weeks post-treatment (Fig. 1). The levels fell below normal range again at 4 weeks post-treatment and remained in the lower range until 9 weeks post-treatment. Unlike HO-776, where a drop in BUN levels was not observed after treatment with 6 mg/kg, a decline in BUN levels was observed at 23 to 28 weeks post-treatment in HO-270 (Fig. 1). Notably, a decrease in BUN levels below the normal range was observed at some point during the study in all the treated animals except HO-784. However, statistical analysis was not performed due to the small number of animals evaluated. None of the horses showed any signs of inappetence, weight loss, or muscle wasting despite the decline in BUN levels.

## Discussion

Treatment of chronically infected horses with the label dose of 2.5 mg/kg of BPQ resulted in rapid suppression of *T. haneyi* within 7 to 8 weeks of treatment until recrudescence of parasitemia occurred. This finding is similar to what was observed in the treatment of *T. equi*-infected horses with BPQ [11], suggesting that BPQ can be used in the management of acute *T. haneyi* infection. This suppression below the limits of detection further suggests that BPQ is more effective in the treatment of *T. haneyi* than imidocarb [6], tulathromycin, and diclazuril [7]. Unlike imidocarb, tulathromycin, and diclazuril that did affect parasite load during infection, BPQ led to rapid abatement of the parasite that was maintained for a minimum of 7 weeks before recrudescence was observed.

The horses were re-treated with an increased dose of 6 mg/kg of BPQ after recrudescence of parasitemia was observed following the initial treatment with 2.5 mg/kg of the drug. This re-treatment was performed to determine whether an increase in BPQ dose would lead to clearance of *T. haneyi*. Unfortunately, parasitemia was maintained after re-treatment with the increased dose, suggesting that the initial treatment with 2.5 mg/kg possibly resulted in the selection of BPQ-resistant parasites. Resistance to BPQ has been demonstrated in the treatment of cattle infected with *T. annulata* [14], and this has been linked to mutations in the cytochrome *b* gene [15–17]. The development of BPQ-resistant *T. haneyi* parasites could also be a possible explanation for the recrudescence of parasitemia observed in this study. We propose two additional explanations for the failure of BPQ in clearing *T. haneyi*. First, drug levels might have been below the theilericidal concentration, or did not persist long enough in the blood, to eliminate all the parasites. Second, a certain parasite subpopulation might have been inaccessible to the drug due to possible sequestration in the animals’ organs. Further studies are therefore warranted to test these hypotheses.

No significant changes were observed in the hematological parameters in the BPQ-treated group, with the exception of BUN, indicating that the drug is relatively safe to use in horses even at a higher dose of 6 mg/kg, as demonstrated previously [11]. The reduced BUN levels after BPQ treatment for extended periods of time, especially in HO-270 and HO-776, could be related to liver dysfunction after BPQ treatment, but this needs to be further investigated in a study using a larger number of animals for statistically significant data.

## Conclusions

In conclusion, 2.5 mg/kg of BPQ has a robust but transient theilericidal effect on *T. haneyi* in chronically infected horses. However, it is evident that the drug at 2.5 mg/kg and 6 mg/kg is ineffective in the clearance of *T. haneyi* infection in persistently infected animals. In the future, pharmacokinetics studies should be considered to determine whether the 6 mg/kg dose is necessarily superior to the 2.5 mg/kg dose. Even though parasite persistence may play a role in immunity in endemic areas of equine piroplasmosis, elimination of *T. haneyi* infection is desirable in non-endemic regions of the disease and in exportation of horses from endemic to non-endemic countries. Therefore, further studies are necessary to evaluate the effectiveness of new drugs in the elimination of *T. haneyi* especially in the persistent phase of infection. Additional investigation is also required to determine whether treatment with BPQ results in emergence of resistant *T. haneyi* strains or whether modifications in the dosage and route of administration could result in clearance of *T. haneyi*.

### Supplementary Information

Supplementary Material 1.

## Data Availability

The original contributions presented in the study are included in the article. Further inquiries can be directed to the corresponding author.

## Abbreviations

PCR: Polymerase chain reaction
rDNA: Ribosomal deoxyribonucleic acid
BPQ: Buparvaquone
HO: Horse (numbers)
PPE: Percentage parasitized erythrocytes
IM: Intramuscular
PBS: Phosphate-buffered saline
fwd: Forward
rev: Reverse
PCV: Packed cell volume
EDTA: Ethylenediaminetetraacetic acid
RBC: Red blood cells
MCV: Mean cell volume
MCH: Mean corpuscular hemoglobin
MCHC: Mean corpuscular hemoglobin concentration
RDW: Red blood cell distribution width
WBC: White blood cells
BUN: Blood urea nitrogen
ALT: Alanine aminotransferase
ALP: Alkaline phosphatase
GGT: Gamma-glutamyl transferase
