# Supplementary material for: Transient efficacy of buparvaquone against Theileria haneyi in chronically infected horses
Source: Parasit Vectors. 2024 Aug 12;17:337. doi: 10.1186/s13071-024-06397-0 (PMC11318256; doi:10.1186/s13071-024-06397-0)
Supplement: Supplementary file 1 — Supplementary Material 1. [file 13071_2024_6397_MOESM1_ESM.docx]

|  | Horse Temperature (°F) | | | | |
| --- | --- | --- | --- | --- | --- |
| Weeks post-treatment | **HO-270** | **HO-364** | **HO-776** | **HO-777** | **HO-784** |
| 0 | 99.3 | 99.7 | 99.4 | 99.2 | 99.0 |
| 3 | 99.2 | 99.5 | 99.9 | 98.6 | 98.6 |
| 4 | 99.8 | 98.8 | 99.0 | 99.1 | 98.5 |
| 5 | 98.0 | 98.5 | 99.0 | 98.4 | 98.6 |
| 6 | 99.3 | 99.2 | 98.6 | 98.6 | 98.6 |
| 7 | 97.7 | 98.3 | 98.0 | 97.9 | 99.0 |
| 8 | 98.8 | 98.9 | 98.1 | 99.4 | 98.9 |
| 9 | 98.3 | 98.0 | 98.8 | 98.4 | 99.0 |
| 10 | 99.4 | 100.4 | 98.8 | 98.1 | 98.7 |
| 11 | 99.0 | 99.3 | 99.4 | 99.1 | 99.0 |
| 12 | 98.3 | 98.9 | 98.0 | 98.7 | 98.3 |
| 13 | 99.1 | 99.1 | 100.0 | 99.3 | 98.6 |
| 14 | 98.1 | 98.9 | 99.4 | 99.1 | 98.6 |
| 15 | 98.6 | 99.4 | 98.5 | 98.5 | 98.1 |
| 16 | 98.3 | 98.8 | 98.5 | 98.6 | 98.6 |
| 17 | 98.6 | 99.5 | 98.3 | 99.0 | 98.8 |
| 19 | 98.7 | 98.7 | 98.3 | 98.6 | 98.1 |
| 20 | 99.4 | 99.4 | 98.0 | 99.1 | 98.7 |
| 21 | 99.4 | 98.7 | 97.9 | 98.6 | 97.9 |
| 22 | 99.0 | 99.9 | 98.1 | 98.7 | 98.1 |
| 23 | 98.8 | 99.1 | 98.2 | 98.6 | 98.1 |
| 24 | 99.8 | 98.8 | 98.1 | 98.8 | 97.8 |
| 25 | 98.9 | 98.3 | 96.9 | 98.8 | 97.4 |
| 26 | 99.4 | 99.8 | 96.7 | 98.7 | 99.2 |
| 27 | 99.5 | 100.0 | 97.9 | 99.4 | 98.0 |
| 28 | 98.6 | 99.5 | 98.7 | 99.2 | 97.6 |
| 29 | 99.8 | 100.0 | 100.0 | 99.5 | 99.4 |
| 30 | 99.1 | 99.1 | 98.1 | 98.6 | 98.6 |
| 31 | 99.1 | 99.4 | 98.7 | 98.9 | 99.1 |
